# Cabergoline Withdrawal Before and After Menopause: Outcomes in Microprolactinomas

**DOI:** 10.1007/s12672-019-00363-4

**Published:** 2019-04-18

**Authors:** Rita Indirli, Emanuele Ferrante, Elisa Sala, Claudia Giavoli, Giovanna Mantovani, Maura Arosio

**Affiliations:** 1 Department of Clinical Sciences and Community Health, University of Milan, Milan, Italy; 2 Endocrinology Unit, Fondazione IRCCS Ca’ Granda Ospedale Maggiore Policlinico di Milano - Padiglione Zonda, Via F. Sforza, 35, 20122 Milan, Italy

**Keywords:** Prolactinoma, Hyperprolactinemia, Menopause, Cabergoline, Dopamine agonist, Recurrence

## Abstract

Natural course of prolactinomas after menopause is not fully elucidated. The aim of this study was to compare recurrence rate after cabergoline withdrawal in premenopausal vs. postmenopausal women with microprolactinoma. Sixty-two women with microprolactinoma treated with cabergoline for at least 1 year and followed for 2 years after drug withdrawal were retrospectively selected. Patients were divided into two groups: 48 patients stopped cabergoline before menopause (“PRE” group), while 14 after menopause (“POST” group). Recurrence was defined by prolactin levels above normal, confirmed on two occasions. Overall, 39/62 women relapsed. Patients who relapsed apparently had higher prolactin before withdrawal (median 216.2, range 21.2–464.3 mIU/L) compared with those in long-term remission (94.3, 29.7–402.8 mIU/L; *p* < 0.05), and the risk of recurrence seemed lower in POST women (4/14, 29%) than in PRE ones (35/48, 73%, *p* < 0.005, OR 0.149, 95% CI 0.040–0.558). However, none of the factors (prolactin before withdrawal, menopausal status, treatment duration, complete adenoma regression) showed a correlation with recurrence risk in multivariate analysis. The best strategy able to optimize CBG treatment and withdrawal’s outcomes is still to be defined in microprolactinomas. Postmenopausal status cannot reliably predict long-term remission, and follow-up is needed also in women of this age.

## Introduction

Microprolactinomas are the most common pituitary adenomas in young women [1], manifesting with oligomenorrhea or amenorrhea, infertility, and galactorrhea. Dopamine agonists, in particular, cabergoline (CBG), are the first-choice treatment, aiming to lower prolactin levels (PRL), reduce adenoma size, and restore gonadal function [2, 3]. Pathogenesis, natural history, and ideal treatment, in terms of duration or criteria for drug withdrawal, are not fully understood for this subtype of pituitary tumor. There is evidence that estrogens can stimulate normal and neoplastic lactotrophs’ growth in vitro and in vivo animal models [4, 5]; however, in humans, evidence is limited to a few studies which have shown a positive effect of the postmenopausal state on the course of disease, with spontaneous and progressive reduction of PRL and adenoma size observed in untreated women, and rare cases of hyperprolactinemia recurrence [6–9]. However, drawing definitive conclusions is difficult for these studies, because patients included were heterogeneous in regard to the etiology of hyperprolactinemia (microprolactinomas vs. macroprolactinomas vs. idiopathic hyperprolactinemia), to the previously employed treatment (surgery vs. medical treatment vs. no treatment) and to the specific drug administered (CBG vs. bromocriptine vs. other dopamine agonists). Also in normoprolactinemic women, some authors showed a trend of spontaneous reduction in PRL after cessation of ovarian activity [10–12], but other studies did not confirm this finding [13].

According to the 2011 Endocrine Society guidelines [2], drug withdrawal may be attempted in women with microprolactinoma when menopause occurs, since the effect of hyperprolactinemia on the hypothalamus-pituitary-gonadal axis is no longer a concern in this period of life, even if long-term follow-up for tumor regrowth is recommended. However, most recent data about the possible direct adverse effects of hyperprolactinemia on bone [14, 15], glucose and lipid metabolism [16], cardiovascular risk profile [17, 18], and quality of life [19–21] may be of concern in postmenopausal women and may deserve consideration when deciding to discontinue dopaminergic treatment.

Which clinical, biochemical, and radiological features, if any, can reliably predict long-term outcome in microprolactinomas remains unclear [22–26]; a better understanding of these aspects is important to help optimize the management of patients with microprolactinoma and may provide an insight in the underlying pathogenetic mechanisms. In order to verify if estrogenic exposure plays a role in microprolactinoma’s recurrence, the aims of the present study were: (1) to analyze clinical, biochemical, and radiological characteristics of patients with microprolactinomas in long-term remission after CBG withdrawal, compared with those experiencing hyperprolactinemia recurrence; (2) to compare recurrence rates after CBG withdrawal in premenopausal and in postmenopausal women; (3) to investigate which factors, including the postmenopausal status, were able to predict the long-term outcomes in both the whole study population and specifically in the postmenopausal group.

## Subjects and Methods

### Inclusion and Exclusion Criteria

Women with a diagnosis of microprolactinoma treated between 1990 and 2015 in our institute were retrospectively investigated in this study. In order to overcome heterogeneity in clinical practice and to select a group of patients whose management had been adherent to current guidelines [2, 3], we established the following selection criteria.

Inclusion criteria:

- female patient with microprolactinoma;
- continuous CBG treatment for ≥ 12 months;
- visible adenoma shrinkage on treatment;
- normal PRL levels before CBG withdrawal;
- the patient had a period of CBG withdrawal and a subsequent follow-up of 24 months.

Exclusion criteria:

- previous surgery and/or radiotherapy of the sellar region;
- multiple endocrine neoplasia (MEN) syndrome, for the well-known peculiarities of prolactinomas’ behavior associated with this condition (greater aggressiveness and resistance to dopamine agonists) [27];
- the patient stopped CBG for pregnancy;
- the patient was taking interfering drugs after CBG withdrawal (e.g., antidepressants, estroprogestinic therapy).

### Definitions and Procedures

The diagnosis of microprolactinoma was based on the presence of typical signs and symptoms (menstrual abnormalities, infertility, galactorrhea), PRL ≥ 530 mIU/L (25 mcg/L), and evidence of a pituitary adenoma < 10 mm in the largest diameter on magnetic resonance imaging (MRI) or computed tomography (CT) scanning of the sellar region with and without contrast enhancement.

At the time of diagnosis, pituitary function was assessed with basal pituitary hormone levels, followed by dynamic testing if clinically appropriate. In all patients, renal failure, primary hypothyroidism, acromegaly, and other secondary causes of elevated PRL (e.g., medications, pregnancy), as well as macroprolactinemia were excluded.

Patients were divided into two groups according to their ovarian status at the moment of CBG withdrawal: patients who stopped treatment before menopause were included in PRE group, while patients who stopped CBG after menopause had occurred were included in the POST group. Menopause was diagnosed after 12 consecutive months of amenorrhea in normoprolactinemic women, in conjunction with raised FSH and low estrogen levels.

We followed up patients for 24 months after drug withdrawal. PRL was assessed at 3, 6, 12, 18, and 24 months. MRI scanning was performed after 1 year in all women, or at any time if a significant raise in PRL was observed. Recurrence of hyperprolactinemia was defined by serum PRL ≥ 530 mIU/L confirmed on two occasions.

The study was approved by the Milan Area 2 Ethical Committee, and informed consent was obtained from all individual participants included in the study.

### Assay Methods

Serum PRL was measured by an electrochemiluminescence sandwich immunoassay (ECLIA, Elecsys Prolactin II, Roche, Mannheim, Germany; for the instrument’s calibration, the 3rd World Health Organization IRP 84/500 was used).

Macroprolactinemia was excluded with polyethylene glycol precipitation (Sigma-Aldrich Inc., Milan, Italy), and results were expressed as percentage of PRL recovery; a recovery ≥ 50% was considered indicative of predominantly monomeric PRL; recovery < 40% was classified as predominantly high molecular weight forms; and recovery between 40 and 50% as indeterminate, and the latter two cases were not included in the study.

### Statistical Analysis

Data were extracted from medical records stored in our department and collected using Microsoft Office Excel software (version 2007). Analysis was performed with IBM SPSS Statistics (version 25).

We performed a Shapiro-Wilk test to define distributions (normal and nonnormal) of quantitative variables. Quantitative parametric variables were expressed as mean ± standard deviation (SD), quantitative nonparametric variables as median and range, and qualitative variables as absolute and relative frequencies. Fisher’s exact test was used to compare number of patients in 2 × 2 contingency tables. Paired or unpaired Student’s *t* test was performed to compare different variables when data were normally distributed. Otherwise, a nonparametric Mann-Whitney test was used. Univariate and multivariate analyses were performed by a logistic regression method. Correlation for continuous variables was assessed by Spearman’s rank correlation test. Values of *p* < 0.05 were considered statistically significant.

## Results

### Patients’ Selection

**Fig. 1:**
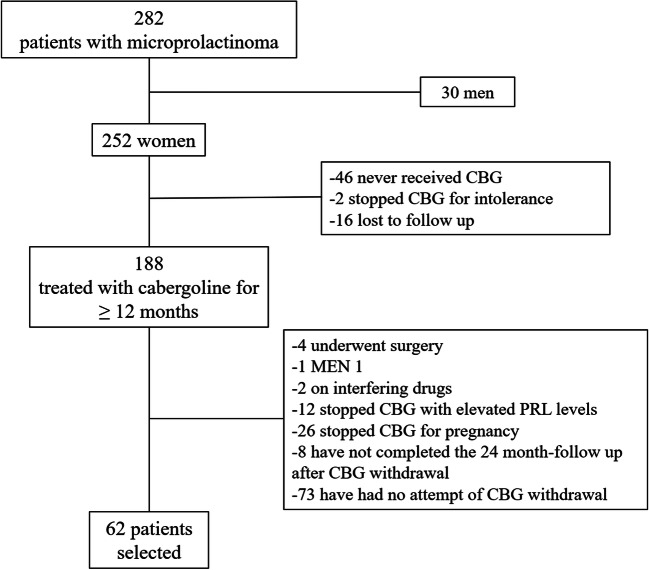
Flow chart showing the process of patients’ selection (CBG = Cabergoline, MEN 1 = Multiple Endocrine Neoplasia syndrome type 1, PRL = Prolactin levels)

Between 1990 and 2015, 282 patients with microprolactinoma, of whom 252 women, were followed at our institute; until 2006, when the Pituitary Society’s guidelines were released, clinical management of patients with microprolactinoma was found to be heterogeneous and often not to fit actual recommendations; in particular, in guiding dopamine agonists’ dose titration and subsequent drug withdrawal, greater importance was given to clinical and radiological response (restoration of regular menses and fertility, disappearance of galactorrhea, adenoma shrinkage), rather than to normalization of PRL levels. In order to overcome these limits, we applied inclusion and exclusion criteria which led to the selection of a homogeneous group of 62 patients (Fig. 1).

In more detail, we excluded patients who had never received CBG (*n* = 46), those who stopped therapy after a few months for intolerance and were consequently treated with bromocriptine or estroprogestinic therapy (*n* = 2), and those who underwent surgery before medical therapy (*n* = 1) or became drug resistant (*n* = 3). Furthermore, 16 women were lost to follow-up, two patients were on potentially interfering drug therapy after CBG withdrawal, and one was diagnosed with MEN 1. Twelve women discontinued CBG after adequate clinical and radiological response but with still elevated PRL levels (median PRL decrease from baseline on CBG therapy 57%, range 42–86%; median PRL levels at withdrawal 806, range 657–1314 mIU/L).

Among the remaining 107 patients, 26 stopped CBG for pregnancy; 8 have recently stopped CBG but have not completed the 24-month follow-up; the others have not stopped CBG yet for different reasons: 13 showed poor treatment compliance and took CBG discontinuously; 21 have been treated for less than 3 years; in 16, no volume reduction was documented during medical treatment and in 9, prolactin levels did not normalize (CBG dose was increased up to 3 mg per week in these patients); 8 patients were desirous of pregnancy and decided to continue CBG until pregnancy was ascertained; 6 are actually undergoing CBG tapering before discontinuation.

### Study Population’s Characteristics and Risk of Hyperprolactinemia Recurrence

The remaining 62 patients represent the study population.

Some women had received one or more different dopamine agonists before CBG.

Complete adenoma regression was documented in 19 out of 62 women before drug withdrawal (31%). For the remaining patients, a median 35% decrease from baseline adenoma size was observed during the CBG treatment (range 12–71%, corresponding to a median variation in maximum diameter of − 1.75 mm, range − 0.7 to − 5 mm).

Prolactin levels’ decrease during the CBG treatment ranged from 69 to 98% of baseline values. Complete clinical and biochemical characteristics are reported in Table 1.

**Table 1:** Clinical and biochemical features of the study patients (column “ALL”) and comparison between women in premenopausal status (PRE) and postmenopausal status (POST) at the time of CBG withdrawal

Group	ALL	PRE	POST	*p* value (PRE vs. POST)
Age at diagnosis, median (range) (years)	29 (15–53)	28 (15–43)	43 (24–53)	< 0.001
Maximum diameter, mean ± SD (mm)	5.7 ± 1.6	5.6 ± 1.6	6.3 ± 1.7	ns
PRL at diagnosis, median (range) (mIU/L)	2056 (933–4537)	2014 (933–4537)	2120 (1102–4070)	ns
Other dopamine agonists prior to CBG (*n*)	21/62^a^	16/48	5/14	ns
Treatment duration, median (range) (years)	3 (1–25)	3 (1–11)	5 (2–25)	0.001
CBG dose per week, median (range) (mg)	0.5 (0.25–1.5)	0.5 (0.25–1.5)	0.5 (0.25–1)	ns
Cumulative CBG dose, median (range) (mg)	78 (13–1300)	52 (13–390)	130 (52–1300)	0.007
Age at withdrawal, median (range) (years)	34 (20–56)	31 (20–49)	51 (46–56)	< 0.001
PRL before withdrawal, median (range) (mIU/L)	161 (21–464)	148 (21–464)	165 (42–403)	ns
Prolactinoma disappearance before withdrawal (*n*)	19/62	14/48	5/14	ns

^a^Sixteen treated with bromocriptine, 1 with lysuride, 2 with dihydroergocryptine, 1 with quinagolide, and 1 with dihydroergocryptine followed by bromocriptine

*PRL*, prolactin levels; *CBG*, cabergoline

Overall, recurrence of hyperprolactinemia was observed in 39 out of 62 women (63%). Twenty out of 39 women relapsed within 3 months after CBG withdrawal and 35/39 (90%) within 12 months. Tumor regrowth was not observed in any of our patients.

Patients who relapsed were not different from those in long-term remission with regard to PRL levels at diagnosis, treatment duration (*p* = 0.074), cumulative CBG dose (*p* = 0.223), previous use of other dopamine agonists, and frequency of adenoma disappearance before withdrawal. The only two features which differentiated patients in the two outcome groups were PRL levels before withdrawal, which were higher in case of hyperprolactinemia recurrence (median 216.2, range 21.2–464.3 mIU/L, vs. median 94.3, range 29.7–402.8 mIU/L for the remission group; *p* = 0.049), and prevalence of postmenopausal women (10/23 in the remission group vs. 4/39 in the recurrence group, *p* < 0.01).

### Comparison of Premenopausal and Postmenopausal Women

Women were then divided into the two groups according to their ovarian status at the moment of CBG withdrawal: 48 patients restored regular menstrual cycles during the CBG treatment and were included in the PRE group; the remaining 14 patients had gone through menopause before CBG withdrawal and were then included in the POST group. The characteristics of the two groups are summarized in Table 1. No woman in the POST group received hormonal replacement therapy after menopause because of the lack of clinical indications.

Patients who stopped CBG after menopause had a significantly lower risk of recurrence compared with patients who stopped before menopause (4/14, 29%, vs. 35/48, 73%; *p* = 0.004, OR 0.149, 95% CI 0.040–0.558).

PRL at diagnosis, previous use of other dopamine agonists, CBG dose per week, PRL before drug withdrawal, and frequency of prolactinoma disappearance at the last MRI were not different in the two groups.

As expected, the PRE group and the POST group were significantly different for age at diagnosis (median 28, range 15–43, vs. median 43, range 24–53 years old respectively, *p* < 0.001) and for age at withdrawal (median 31, range 20–49, vs. median 51, range 46–56 years old respectively, *p* < 0.001). Postmenopausal patients were treated for a longer time (median 5, range 2–25 years) than premenopausal ones (median 3, range 1–11; *p* = 0.001), and consequently received higher cumulative CBG doses (median 130, range 52–1300 mg, vs. median 52, range 13–390 mg in PRE patients, *p* = 0.007).

### Predictive Factors for Long-Term Outcomes

We had previously found that treatment duration was not different in the two outcomes (hyperprolactinemia recurrence vs. long-term remission) in our population. However, in order to further investigate whether longer treatment duration and higher cumulative CBG doses could have influenced the higher remission rate in postmenopausal women, we performed binomial logistic regression for these two independent variables. Univariate analysis confirmed lack of association between hyperprolactinemia recurrence and both treatment duration (*p* = 0.226) and cumulative CBG dose (*p* = 0.283). Additionally, no association was found between tumor disappearance before withdrawal and probability of long-term remission (*p* = 0.427) in univariate analysis, as well.

Given that the beneficial effects of menopause on microprolactinomas are supposed to be due to a reduction of circulating estrogens, we tested whether a correlation exists between estradiol and prolactin. Median estradiol levels resulted 35 (range 0–95) pg/mL before CBG treatment, 33 (0–477) pg/mL on therapy, and 26 (5–217) pg/mL after withdrawal. Again, we failed to find a correlation between the two hormones’ levels at the three time points (before treatment: rho = − 0.258, *p* = 0.318; on CBG treatment: rho = − 0.126, *p* = 0.681; after withdrawal: rho = 0.112, *p* = 0.703).

Finally, we tested which factors could predict recurrences in microprolactinomas by a linear logistic regression analysis; when multivariate analysis was performed, correlation of microprolactinomas’ outcomes with PRL levels at withdrawal (*p* = 0.054) and with the postmenopausal status (*p* = 0.054) was not confirmed.

### Postmenopausal Patients

Twenty-eight percent of women who stopped CBG after menopause relapsed. In this group of women, PRL at diagnosis and before withdrawal, treatment duration, cumulative CBG dose, previous use of other dopamine agonists, and frequency of adenoma disappearance before withdrawal were not different whether patients relapsed or remained in long term remission (data not shown).

## Discussion

This study retrospectively analyzes the risk of hyperprolactinemia recurrence in microprolactinomas after suspension of CBG, particularly comparing the effects of stopping the treatment before and after menopause.

Overall, hyperprolactinemia recurrence occurred in 63% of women, which fits the very variable data reported in the literature (from 7 to 69% [22, 23, 28–32]); given the well-known stimulating effect of estrogens on prolactin secretion in vivo and in vitro, we investigated if a physiological hypoestrogenic state-like menopause could impact the success of CBG withdrawal in microprolactinomas; however, neither the menopausal status at withdrawal nor the other variables tested (treatment duration, PRL before withdrawal, complete adenoma regression) were associated with long-term outcomes in our population. Furthermore, no association was found between the prolactin and estradiol levels.

In literature, many clinical, biochemical, and radiological characteristics have been investigated as potentially predictive factors for long-term remission in prolactinomas, like treatment duration, complete tumor regression before withdrawal, and nadir PRL reached during dopamine agonist treatment [22, 25, 26, 33–35], and some were included by most recent guidelines as criteria for drug withdrawal [2, 3].

The lack of association that we found between CBG withdrawal’s outcomes and complete tumor regression, treatment duration, or cumulative CBG doses is concordant with other previously published research [23, 25, 33]. In particular, given the unclear role of treatment duration on microprolactinomas’ outcomes, we decided to include in the present study patients treated with CBG for at least one year, although most recent guidelines [2, 3] recommend a 2–3-year treatment before drug withdrawal. As stated above, we then verified that treatment duration did not impact the risk of hyperprolactinemia recurrence in our population.

Two meta-analysis on this subject have recently been released [22, 34]. Hu et al. [22] found a hyperprolactinemia recurrence rate of 60% in microprolactinomas treated with CBG. Continuation of the CBG treatment for longer than 2 years was not associated with higher success rates, similar to our results on treatment duration. However, Xia et al. in their meta-analysis [34] affirmed the opposite, since they found a higher remission rate when treatment was continued for more than 24 months. Furthermore, both meta-analysis found that a significant tumor volume reduction (i.e., ≥ 50% decrease from baseline) was relevant to long-term outcome. However, authors did not differentiate microprolactinomas and macroprolactinomas in this respect. Additionally, in our study we did not focus on the degree of tumor shrinkage, but only compared complete tumor regression vs. adenoma persistence, so these results are not easily comparable. Interestingly, both meta-analyses found that a low CBG maintenance dose (i.e., ≤ 0.5 mg per week) is associated with a higher remission rate.

According to current literature [2, 6–8, 19, 36], dopamine agonist withdrawal can be considered safe when menopause occurs, with low rates of hyperprolactinemia recurrence; however, only a few studies have specifically focused on prolactinomas’ outcomes after menopause. Karunakaran et al. [7] found that, among menopausal women, 45% normalized PRL off treatment, compared with only 7% of women with preserved ovarian function; however, patients in Karunakaran’s study were quite heterogeneous in regard to hyperprolactinemia etiology (idiopathic vs. microprolactinoma) and to previous treatment (surgery vs. dopamine agonists vs. no treatment); in fact, among postmenopausal women, only 7/11 had received dopamine agonists, while two had undergone surgery. Touraine et al. [6] described a small group of 4 women with microprolactinoma whose PRL spontaneously reduced off treatment after menopause. Mallea-Gil et al. followed 22 patients with microprolactinoma for a period between 4 and 192 months after CBG or bromocriptine suspension and found that only two patients needed to restart treatment for increasing PRL levels; among the remaining 20, a spontaneous reduction of PRL and a high rate of tumor disappearance at MRI were found [8]. In a recently published study from UK [9], Santharam et al. found that among 16 postmenopausal women who stopped medical treatment after menopause with normalized prolactin levels, recurrence rate was significantly lower than that in premenopausal ones and comparable with that observed in our study, 31%. In their study, no difference in terms of recurrence rate was observed according to the adenoma size or drug used (bromocriptine vs. cabergoline). Interestingly, an increase in adenoma volume after treatment withdrawal was reported in two postmenopausal patients. PRL at 6–12 months off treatment was the only predictive factor for long-term remission.

In our study, no factor could explain differences in the outcomes of CBG withdrawal between premenopausal and postmenopausal women. In fact, even though postmenopausal women had been treated with CBG for a longer time than premenopausal patients, the treatment duration did not show any statistical association with long-term outcome, as stated above. The two groups of women were comparable for all other characteristics, so one may suppose that the favorable outcome observed in postmenopausal women could be due to hypoestrogenism. Estrogen is a very well-known stimulus for PRL synthesis and secretion. In fact, microprolactinomas are most frequently found in young women in their fertile age (20–50 years old) [19]; in animal models, ovariectomy causes a reduction in pituitary lactotrophs’ size and number and a reduction in circulating prolactin levels; these effects can be reversed by estrogen administration [4]. In addition, it was shown that anti-estrogens reduce PTTG expression in human pituitary tumors in vitro and suppress tumor growth in rats in vivo, concomitantly with reduced PRL secretion [5]. However, in humans, evidence is limited; in our study, between one quarter and one-third of postmenopausal women relapsed and PRL showed no correlation with estradiol levels, thus suggesting that other factors may stimulate prolactinomas’ growth and secretion; for instance, whether other neuro-endocrinological systems and molecules, which may eventually modify after menopause, play a role in lactotrophs’ modulation is not known. Pathogenetic mechanisms for microprolactinomas, including genetics, signaling pathways, and local stimuli, are still far from being fully understood. In our opinion, this may partly explain why, despite many efforts to identify predictive factors for long-term outcomes in these adenomas, clinical data are largely discordant and definitive conclusion can be hardly drawn yet.

The clinical implications of hyperprolactinemia recurrence in menopause are not known. In fact, gonadal dysfunction is no more a concern after menopause, so microprolactinomas should not deserve dopaminergic treatment unless tumor regrowth is observed. However, possible effects of hyperprolactinemia on bone loss [14, 37], insulin resistance, dyslipidemia [16, 38, 39], cardiovascular risk [17, 18, 40], and quality of life [19–21] could be of some importance in this group of women; long-term follow-up is recommended at least until all these aspects are fully elucidated.

Our study has several strengths. We focused on current management strategies for prolactinomas, including only patients treated with CBG and excluding those who had undergone surgical treatment or radiotherapy, that are nowadays limited to resistant prolactinomas; in this way, we tried to select a homogeneous group of women with regard to hyperprolactinemia etiology and treatment employed, and this represents a novelty compared with previous studies on this subject. On the other side, our study has some limitations: first, its retrospective design. The sample size is quite small, however comparable with that of previously published research on prolactinomas in postmenopausal women, and we recognize that this could have partly influenced the negative results. Another limitation is the short follow-up time; even if it is well-known that most patients relapse within the first year after drug withdrawal [25, 33], longer time may be needed to better understand prolactinoma’s natural course.

In conclusion, CBG is an effective treatment for microprolactinomas, but the best strategy in order to optimize outcomes after drug withdrawal is still to be defined. Despite the supposed stimulatory effect of estrogens on microprolactinomas, a physiological hypoestrogenic state-like menopause is not able to predict remission and nearly a third of postmenopausal women relapse. For this reason, we believe that, if a trial of CBG withdrawal is attempted, monitoring for hyperprolactinemia recurrence is necessary also in older women.
